# An Orally Active Allosteric GLP-1 Receptor Agonist Is Neuroprotective in Cellular and Rodent Models of Stroke

**DOI:** 10.1371/journal.pone.0148827

**Published:** 2016-02-10

**Authors:** Huinan Zhang, Yunhan Liu, Shaoyu Guan, Di Qu, Ling Wang, Xinshang Wang, Xubo Li, Shimeng Zhou, Ying Zhou, Ning Wang, Jingru Meng, Xue Ma

**Affiliations:** 1 Department of Pharmacology, School of Pharmacy, the Fourth Military Medical University, Xi’an, China; 2 School of Nurse, the Fourth Military Medical University, Xi’an, China; 3 Department of Health Statistics, Faculty of Preventative Medicine, the Fourth Military Medical University, Xi’an, China; Indian Institute of Integrative Medicine, INDIA

## Abstract

Diabetes is a major risk factor for the development of stroke. Glucagon-like peptide-1 receptor (GLP-1R) agonists have been in clinical use for the treatment of diabetes and also been reported to be neuroprotective in ischemic stroke. The quinoxaline 6,7-dichloro-2-methylsulfonyl-3-N-tert- butylaminoquinoxaline (DMB) is an agonist and allosteric modulator of the GLP-1R with the potential to increase the affinity of GLP-1 for its receptor. The aim of this study was to evaluate the neuroprotective effects of DMB on transient focal cerebral ischemia. In cultured cortical neurons, DMB activated the GLP-1R, leading to increased intracellular cAMP levels with an EC_50_ value about 100 fold that of exendin-4. Pretreatment of neurons with DMB protected against necrotic and apoptotic cell death was induced by oxygen-glucose deprivation (OGD). The neuroprotective effects of DMB were blocked by GLP-1R knockdown with shRNA but not by GLP-1R antagonism. In C57BL/6 mice, DMB was orally administered 30 min prior to middle cerebral artery occlusion (MCAO) surgery. DMB markedly reduced the cerebral infarct size and neurological deficits caused by MCAO and reperfusion. The neuroprotective effects were mediated by activation of the GLP-1R through the cAMP-PKA-CREB signaling pathway. DMB exhibited anti-apoptotic effects by modulating Bcl-2 family members. These results provide evidence that DMB, a small molecular GLP-1R agonist, attenuates transient focal cerebral ischemia injury and inhibits neuronal apoptosis induced by MCAO. Taken together, these data suggest that DMB is a potential neuroprotective agent against cerebral ischemia.

## Introduction

Ischemic stroke is a leading cause of adult morbidity and mortality worldwide with very limited treatment options [1]. Tissue plasminogen activator (tPA), which acts by dissolving clots after intravenous injection, is the only Food and Drug Administration (FDA)-approved therapy for the treatment of stroke. However, only about 2% of stroke patients are eligible for tPA treatment. This lack of treatment options highlights the need for new therapeutics aimed at the prevention and treatment of ischemic stroke.

Glucagon-like peptide 1 (GLP-1) is a 30-amino acid peptide secreted from the L-cells of the small intestine [2]. GLP-1 exerts its effects by binding to GLP-1 receptor (GLP-1R), a member of the class B family of seven transmembrane G protein-coupled receptors (GPCRs) [3]. GLP-1R is widely expressed in the brain, and GLP-1R activation mediates neuroprotection in animal models of Alzheimer’s, Parkinson’s, Huntington’s, stroke and other degenerative diseases [4–7]. GLP-1 and analogues cross the blood-brain barrier, protect memory formation or motor activity, enhance neurogenesis, reduce apoptosis, protect neurons from oxidative stress, and reduce chronic inflammation response [6, 7]. Therefore, GLP-1R is considered to be an effective and promising therapeutic target for nervous system diseases.

The GLP-1R stimulates cAMP by coupling to the Gαs subunit. Serine protease dipeptidyl peptidase-IV rapidly degrades GLP-1 in plasma, resulting in a half-life of only about 1 min [8]. Thus, GLP-1 analogs with a longer plasma half-life were developed. Currently, five long-lasting GLP-1 analogs have been approved by the FDA and the European Medicines Agency (EMA) for the treatment of Type 2 diabetes (T2D): exenatide twice-daily (Byetta^®^, Amylin/Lilly) and exenatide once-weekly (Bydureon^®^, Amylin/Lilly) are based on exendin-4; liraglutide once daily (Victoza^®^, Novo Nordisk), Lixisenatide once daily (Lyxumia^®^, Sanofi) and Albiglutide once-weekly (Tanzeum^®^/Eperzan^®^, GSK) are based on the structure of native GLP-1. Several additional GLP-1 mimetics, including Dulaglutide once-weekly (Trulicity^®^, Lily), semaglutide once-weekly (NovoNordisk) and others are in various stages of clinical trials [9]. These GLP-1 mimetics possess favorable pharmacokinetic properties, including reduced frequency of injections and improved glycemic control throughout the day [9, 10]. Recent studies have reported that these GLP-1 analogs, such as exendin-4 and liraglutide, exert neuroprotective effect on ischemic stroke [4, 5, 11–15]. Because hyperglycemia is one of the leading risk factors for ischemic cerebrovascular diseases [16], it provided a potential clinical use of GLP-1R agonists for the treatment of stroke in T2D patients or individuals at high risk to suffer from a stroke (e.g. pretreatment strategies). Unfortunately, GLP-1 and its analogues require administration by subcutaneous or intravenous injection. Hence, the development of an orally active non-peptide small molecular weight GLP-1R agonist is desirable.

**Fig 1:**
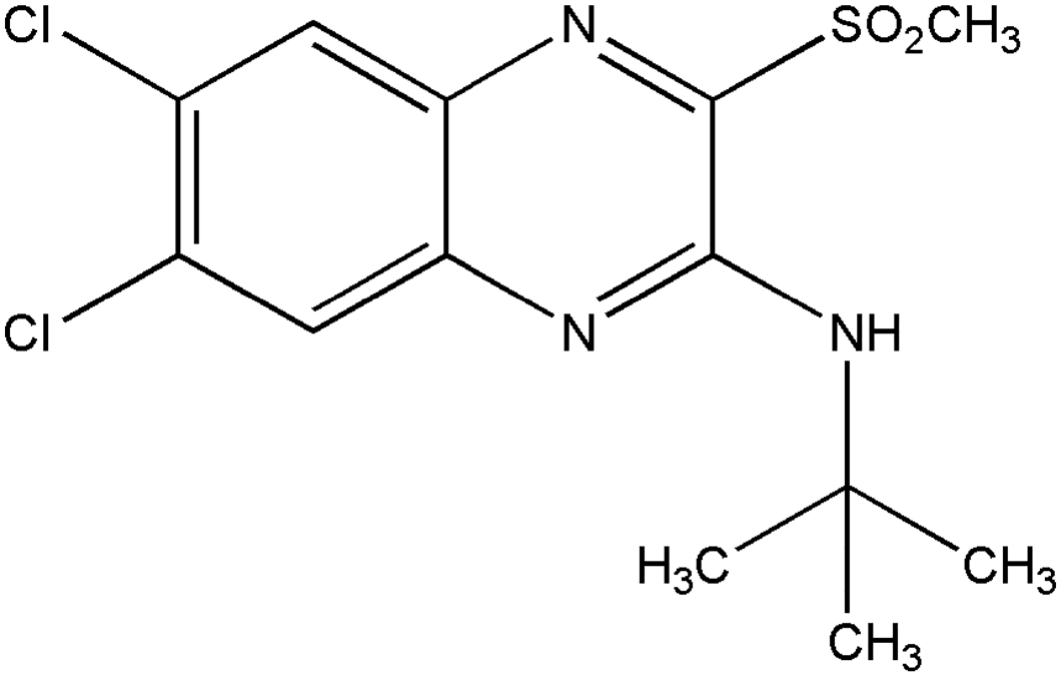
Structure of DMB, molecular formula C_13_H_15_Cl_2_N_3_O_2_S.

Recently, a series of substituted quinoxalines and a cyclobutane derivative were reported to serve as a scaffold for nonpeptide GLP-1R agonists. One such candidate, 6,7-dichloro-2-methylsulfonyl-3-N-tert-butylaminoquinoxaline (DMB, Fig 1), is a quinoxaline compound that acts as an agonist and allosteric modulator of the GLP-1R. DMB has the potential to increase the affinity of GLP-1 for its receptor and stimulate cAMP, which potentiates glucose-dependent insulin release in β-cells. However, the efficacy of DMB on the GLP-1R in the brain has not been previously reported. In the current study, we evaluated the neuroprotective effects of DMB as a small molecular GLP-1R agonist on transient focal ischemia induced cerebral damage with an oxygen-glucose deprivation (OGD) model in cultured neurons and MCAO and reperfusion model in mice.

## Materials and Methods

### Materials

DMB was purchased from Calbiochem (Nottingham, UK). Neurobasal, B27 and fetal bovine serum were purchased form Invitrogen (Calsbad, CA, USA). Antibodies against PKA, p-CREB, CREB, Bax, Bcl-2 and GLP-1R as well as the goat anti-mouse and anti-rabbit IgG secondary antibodies were purchased from Santa Cruz Biotechnology, Inc. (Santa Cruz, CA, USA). Antibodies against β-actin, exendin-4 and exendin (9–39) were purchased from Sigma-Aldrich (Saint Louis, MO, USA) along with the Hoechst 33258, and PI. The plasma insulin ELISA kit was purchased from the Millipore Corporation (Billerica, MA, USA); cAMP parameter assay kit and TUNEL staining in situ cell death detection kit were purchased from Roche (Minneapolis, MN, USA) and the LDH detection kit was purchased from Jiancheng Inc. (Nanjing, Jiangsu, China). CeneChem Co., Ltd. (Shanghai, China) prepared the pGV118 lentiviral vector encoated shRNA targeting the GLP-1R.

### Primary mouse cortical neuronal culture

Primary cortical neurons were taken from 18-day-old neonatal C57BL/6 mice. Neurons were dissociated, minced, and trypsinized with 0.125% trypsin and then seeded into plates of 6 or 96 wells using DMEM containing 20% fetal bovine serum and at a density of approximately 2,500 cells/mm^2^. The culture medium was then replaced by neuron-basal medium supplemented with 2% B27 at 37°C. Neurons were cultured for 7 days and then used for further experiments.

### Determination of cAMP

Primary culture neurons were seeded into 96 well culture plates for 7 days. LV-shGLP-1R (10^5^ TU/ml) was added 4 days and exendin (9–39) (1 μM) was added 30 min prior to the addition of DMB (10^−7^–10^−4^ μM) or exendin-4 (10^−10^–10^−6.5^ μM) to the culture wells. Cells were allowed to incubate for 0–30 min following treatment with DMB or exendin-4. A cAMP assay kit was then used to determine cAMP levels according to the manufacturer’s instructions. All data were subsequently normalized to the response of 1 mM GLP-1.

### Oxygen-glucose deprivation (OGD)

OGD on primary neuronal cultures was performed as previously reported [17]. Briefly, cultured primary cortical neurons were washed with PBS 3 times, then placed in glucose-free EBSS buffer and stored in an anoxic chamber filled with 95% N_2_ and 5% CO_2_ for 6 h. The culture medium was then replaced by fresh neuron-basal medium supplemented with 2% B27 and the neurons were returned to normoxic conditions for reoxygenation for 12 h.

### Cell treatment paradigm

LV-shGLP-1R (10^5^ TU/ml) was added 4 days and exendin (9–39) (1 μM) was added 30 min prior to the addition of DMB (1 μM) to the cell culture medium. One hour later, cells underwent OGD. Neurons in the OGD group were treated with an equal volume of DMSO; the normoxic group did not receive any treatment.

### Cell viability assay

The cell survival and death rates of the neurons were determined using an MTT and LDH assay 24 h after OGD injury. For the MTT assay, MTT was added at a final concentration of 0.5 mg/ml and incubated at 37°C for 4 h. The culture medium was then replaced with DMSO and the optical density was determined at 490 nm. LDH activity was measured with an LDH assay kit according to the manufacturer’s instructions. The optical density was measured at a wavelength of 490nm. Neurons maintained under normoxic conditions were defined as 100% viable. The viability of neurons exposed to OGD conditions is expressed as a percentage compared to cells under normoxic conditions.

### Hoechst/PI double staining

Hoechst/PI staining was performed 24 h after OGD. Briefly, Hoechst 33258 and PI were added into the culture medium at a final concentration of 10 μg/ml for 15 min and then fixed with formaldehyde for 30 min. Neurons were observed under a fluorescent microscope (Olympus BX61, Japan). Hoechst was excited at 340 nm and PI was excited at 620 nm.

### Experimental animals and drug treatment

Adult male C57BL/6 mice weighing 18–22 g were used in our research. The Animal Care and Use Committee of the Fourth Military Medical University approved all animal protocols. Mice were maintained in a climate-control room with a 12 h light-dark cycle with access to food and water ad libitum throughout the experimental period. All surgery was performed under anesthesia, every effort was made to minimize the number of animals used and their suffering. After acclimation to the environment for 7 days, mice were treated with DMB (5 μmol/kg, orally) and/or exendin-4 (10 nmol/kg, i.p.) 30 min prior to MCAO.

### Determination of blood glucose and plasma insulin levels

DMB (5 μmol/kg) was orally administrated to C57BL/6 mice and blood samples collected at 0, 30, 60 and 120 min after administration. The plasma insulin level was determined with an ELISA kit. The blood glucose level was determined using a glucometer (Roche Diagnostics, Mannheim, Germany).

### Animal model of focal cerebral ischemia

The middle cerebral artery occlusion procedure was conducted as previously described [18]. Briefly, the animals were anesthetized with 1% pentobarbital sodium. The right carotid region was exposed and a 6–0 nylon filament inserted through the external carotid artery and gently advanced 11 mm to occlude the middle cerebral artery (MCA) for 60 min. Reperfusion was performed by withdrawing the nylon monofilament.

### Neurological deficits score assay and infarct volume measurement

The neurological deficit scores were evaluated in a blinded fashion 24 h after MCAO and reperfusion based on Bederson’s score standard [19]. Specifically, grade 0 represents no observed deficit; grade 1 represents flexion to contralateral torso and forelimb; grade 2 represents a decreased resistance to lateral push but without circling; grade 3 represents leaning to affected side; and grade 4 represents no spontaneous locomotor activity. Dead animals were excluded in the scoring scale. After evaluation of neurological deficits, the infarct volume was measured by TTC staining, which has been widely used for accurately reflecting the extent of irreversible ischemic damage in cerebral tissues [20]. Briefly, the brain was removed and frozen at -20°C for 30 min and then sectioned into slices 1 mm thick. The slices were then incubated in a 1.5% TTC solution at 37°C for 30 min and fixed in paraformaldehyde. Samples were digitally photographed and the percentage of the infarct volume with respect to the total brain volume was quantified with Photoshop CS4.

### Silencing of the GLP-1R by LV-shGLP-1R

A specific sequence of shRNA targeting to GLP-1R mRNA (GenBank Accession No. NM_021332.2, 608–628) was constructed into a pGV118 lentiviral vector. For culture neurons, LV-shGLP-1R was dissolved in an enhanced infection solution and added to medium 4 days prior to drug treatment at the titre of 10^5^ TU/ml for 12 h. For C57 mice, LV-shGLP-1R was stereotaxically delivered into the ipsilateral ventricle (bregma: -0.4 mm, dorsoventral: 0.7 mm, lateral: 1.8 mm) 7 days before MCAO injury as previously described [21]. Silencing efficiency was assayed by detecting GLP-1R levels in neurons or the ipsilateral ischemic penumbra area of C57 mice (S1 Fig).

### TUNEL staining

TUNEL staining was performed 24 h after MCAO as previously described [22]. Mice were deeply anesthetized and perfused with cold saline followed by 4% paraformaldehyde. The ipsilateral ischemic penumbra section (20 μm) was selected for TUNEL staining. TUNEL staining was performed using an in situ cell death detection kit performed according to the manufacturer’s protocol. The stained sections were photographed with a confocal fluorescent microscope. The apoptotic neurons were counted and results are expressed as TUNEL positive cells per mm^2^.

### Western Blot

Protein samples in cultured neurons were collected 7 days after plating. The protein samples were extracted from the ipsilateral ischemic penumbra area 24 h after MCAO or 7 days after stereotaxically injecting LV-shGLP-1R in the mice. After homogenization in cold lysis buffer containing 1 nM PMSF, protein levels were determined using bichioninic acid. A 20 μg protein sample was loaded onto an SDS page gel and transferred to polyvinylidene fluoride membranes and blocked in TBS containing 5% nonfat milk for 2 h at room temperature. The membranes were incubated with anti-GLP-1R (1:1,000), anti-PKA (1:1,000), anti-p-CREB (1:500), anti-CREB (1:500), anti-Bax (1:500), anti-BCL-2 (1:500; Santa), or anti-β-actin (1:5,000) overnight at 4°C, then incubated with goat anti-mouse or anti-rabbit IgG secondary antibodies (1:10,000) for 1 h. The blots were visualized and quantified with densitometry.

### Statistical Analysis

Statistical analysis was performed using SPSS 13.0. All data except for neurological scores are expressed as mean±SD and differences between groups were analyzed by one-way ANOVA followed by a Dunnett T-test. The neurological scores were analyzed with the Kruskal–Wallis test followed by the Mann-Whitney U-test with the Bonferroni correction. Significant differences were accepted if the *p* value was < 0.05.

## Results

### 1. The GLP-1R was present in cortical neurons and activated by DMB

**Fig 2:**
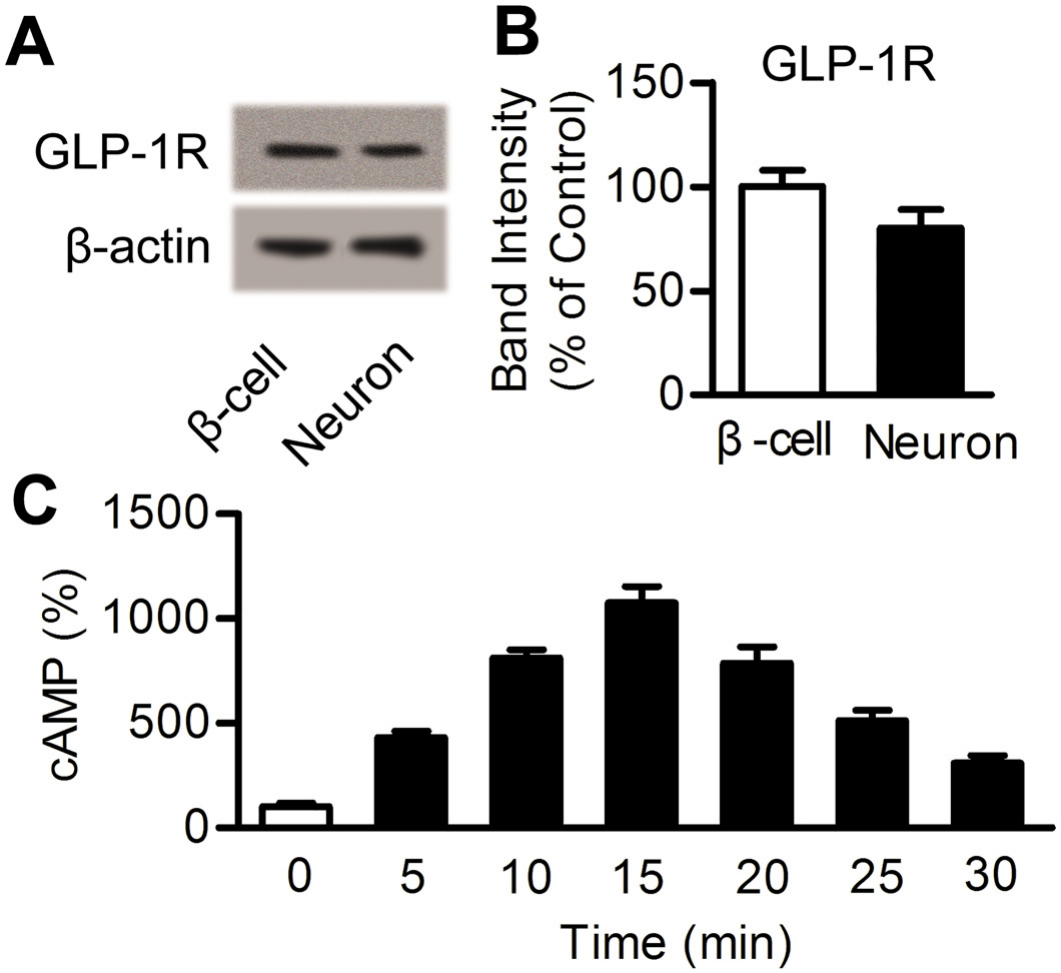
DMB activates the GLP-1 receptor in cortical neurons. (A) Western blot showing GLP-1R expression in neurons and β-cells. (B) Bar graph reflecting the GLP-1R expression in each group. Data are presented as percentage of β-cells. n = 5 (C) cAMP stimulation by DMB (1 μM) in neurons and detected 0–30 min after incubation. Data are expressed as percentage of 0 min. n = 6.

A Western blot was used to detect the presence of the GLP-1R in both cortical neurons and β-cells, which were used as a positive control (Fig 2A and 2B). In cortical neurons incubated with DMB (1 μM), we observed a rapid, transient elevation of intracellular cAMP levels. The cAMP reached its peak level after 15 min and then returned toward baseline at 30 min (Fig 2C). These data demonstrate that the GLP-1R receptor is present in primary cortical neurons and can be activated by DMB.

### 2. The mechanism of GLP-1R receptor activation by DMB is different than exendin-4

**Fig 3:**
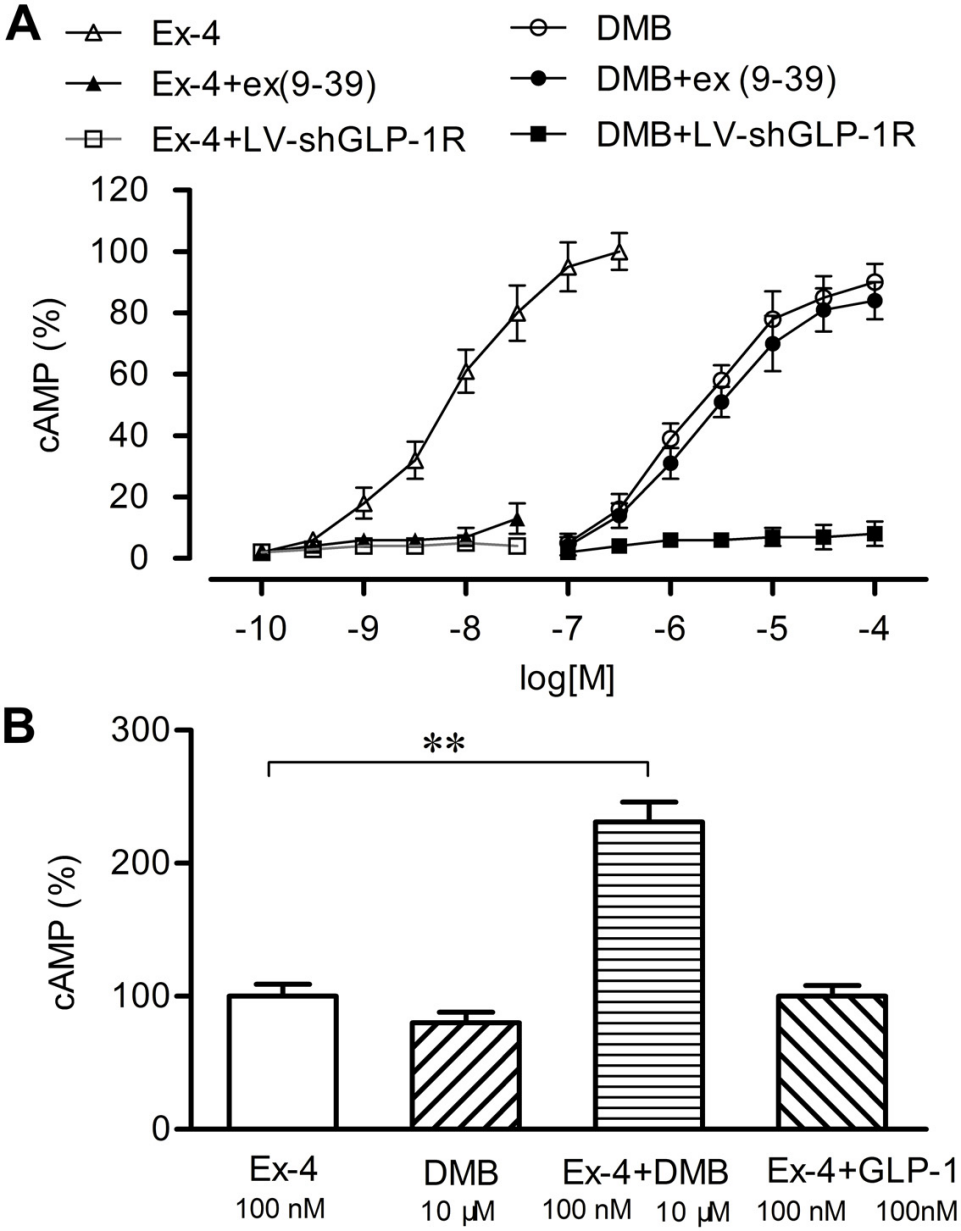
DMB and exendin-4 activate the GLP-1R in neurons by different mechanisms. (A) DMB and exendin-4 show different responses in the presence of exendin (9–39) or LV-shGLP-1R. (B) cAMP was stimulated in neurons by exendin-4 (100 nM), DMB (10 μM) and/or GLP-1 (100 nM). cAMP was detected 15 min after incubation. ex (9–39): exendin(9–39), data are expressed as a percentage of cAMP stimulation induced by 1 mM GLP-1. ** *p* < 0.01.

We compared the effect of DMB on the GLP-1R in neurons with exendin-4, a long lasting peptide agonist that has been used clinically. The results show that DMB mediated cAMP generation after a 15 min incubation period with a 90% efficacy relative to stimulation by 1 mM GLP-1; DMB displayed an EC_50_ value about 100 fold compared with exendin-4 (Fig 3A). To further evaluate the mechanism, we exposed the cells to exendin (9–39) (1 μM), an orthosteric competitive antagonist [23, 24]. The results show that exendin (9–39) resulted in nearly complete inhibition of the cAMP generation stimulated by exendin-4, but had no effect on the cAMP generated by DMB (Fig 3A). However, LV-shGLP-1R (10^5^ TU/ml) diminished the stimulation of cAMP generated by both exendin-4 and DMB (Fig 3A). These results indicate that the interaction of DMB with the GLP-1R in neurons is through a different mechanism than exendin-4. DMB reportedly exhibits agonist and positive allosteric modulator activity at the GLP-1R without competing with exendin-4 for receptor binding [25]. Thus, the effect of DMB on neurons in the presence of exendin-4 was evaluated. Results indicate that co-incubation of DMB (10 μM) with exendin-4 (100 nM) further stimulates cAMP generation; however, the combination of GLP-1 (100 nM) and exendin-4 (100 nM) did not exert a similar effect (Fig 3B). These results indicate that DMB and exendin-4 induce GLP-1R activation in an additive manner in neurons.

### 3. DMB decreased OGD induced death of primary cultured neurons

**Fig 4:**
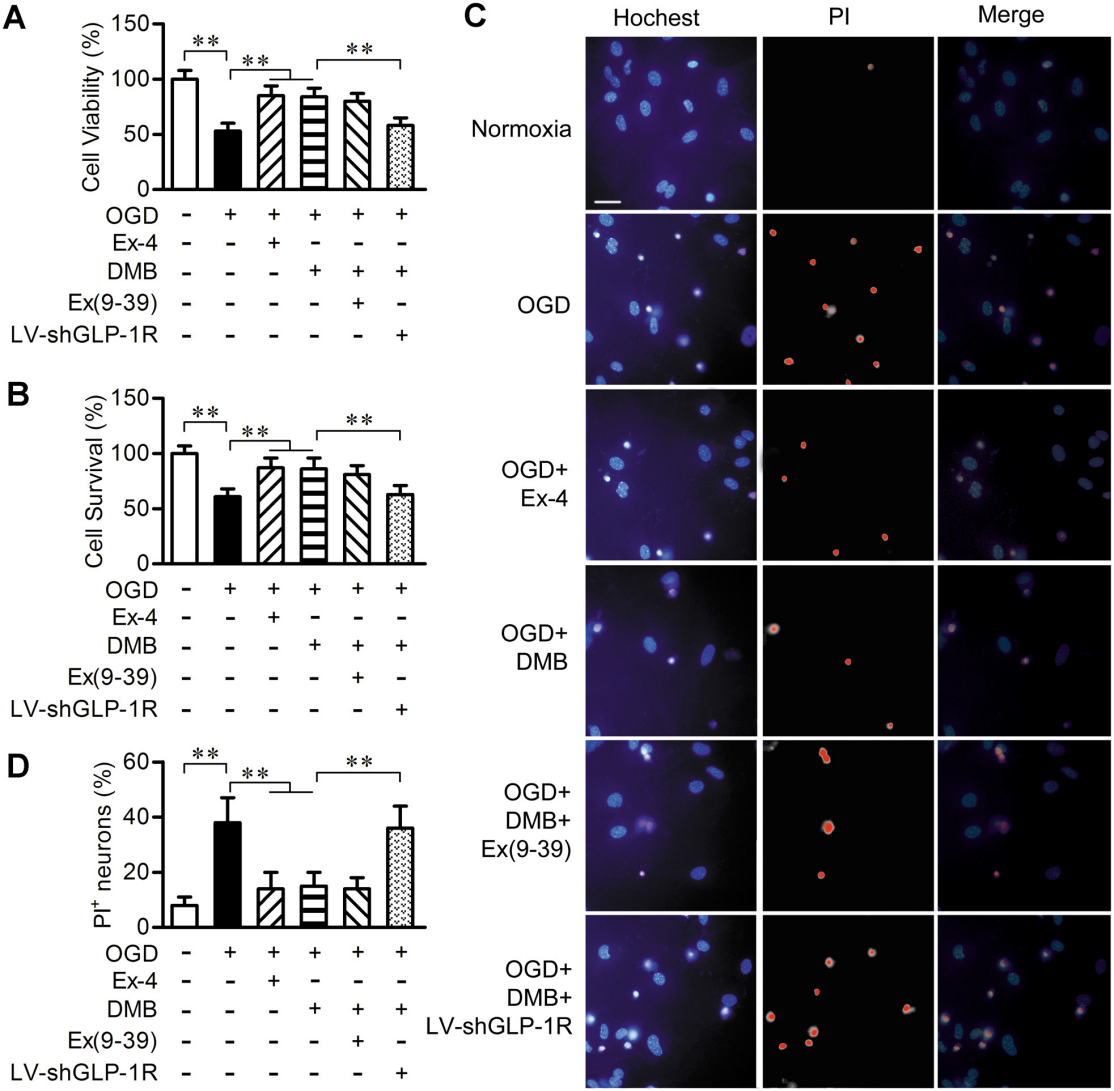
DMB increased cortical neuron survival in OGD and reperfusion. (A) Quantification of cell viability in each group by MTT. n = 6. (B) Quantification of cell survival in each group by LDH. n = 6. (C) Image of Hoechst and PI staining in cortical neurons. Scale bar = 25 μm. (D) Bar graph reflects percentage of apoptotic neurons following OGD or normoxia. Six different fields were counted. ** *p* < 0.01.

OGD and reperfusion is an in vitro model that mimics the condition of ischemia-reperfusion injury [26]. Therefore, the action of DMB against cerebral ischemia *in vitro* was studied in this model. Cell viability assays by MTT and LDH showed that OGD and reperfusion injury lead to a decrease in neuronal survival compared with cells under normoxic conditions. Pretreatment with DMB (1 μM) and exendin-4 (10 nM) significantly increased the cell survival rate compared with the OGD only group. The GLP-1R peptide antagonist exendin (9–39) (1 μM) had no impact on the neuroprotective efficiency of DMB. However, silencing the GLP-1R by LV-shGLP-1R (10^5^ TU/ml) almost totally attenuated this effect (Fig 4A and 4B). As expected, the results of Hoechst 33258/PI double staining showed that the necrotic and apoptotic neurons caused by OGD and reperfusion were reversed by DMB and exendin-4. LV-shGLP-1R (10^5^ TU/ml) but not exendin (9–39) (1 μM) blocked the beneficial effects of DMB (Fig 4C and 4D).

### 4. DMB had no impact on plasma insulin and glucose levels in non-diabetic mice

**Fig 5:**
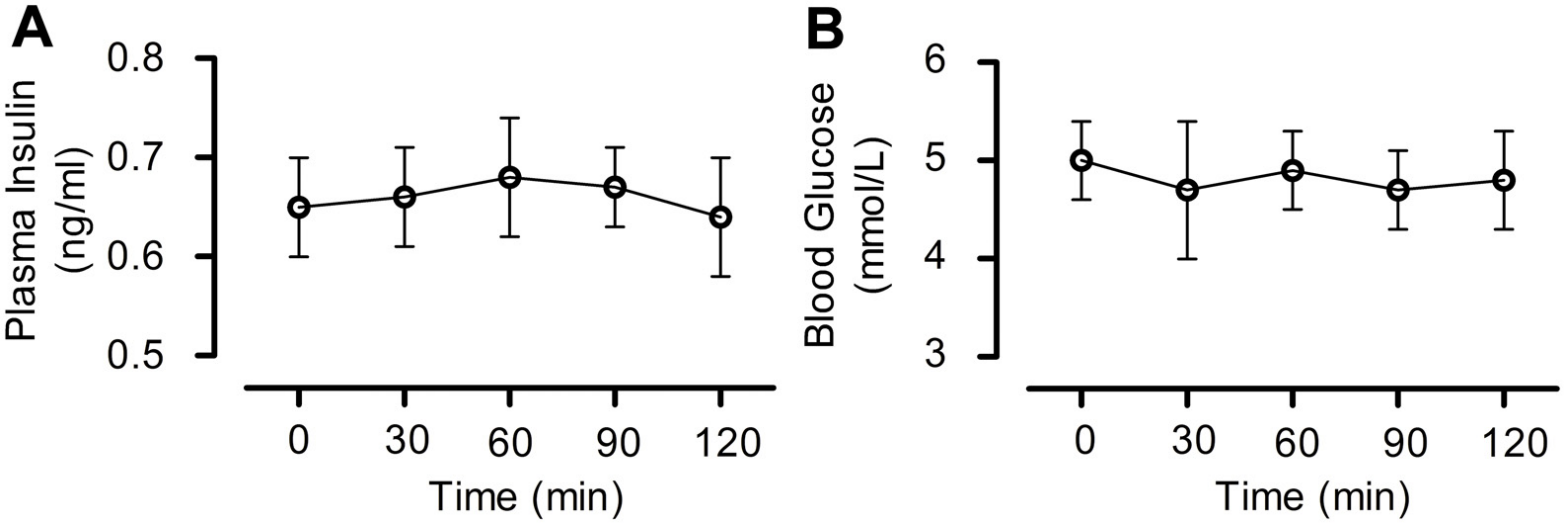
Effects of DMB on plasma insulin and glucose levels. DMB (5 μmol/kg) was orally administered to C57BL/6 mice. Insulin and glucose levels were detected at 0, 30, 60, 90 and 120 min after administration. (A) Plasma insulin levels. (B) Blood glucose levels. n = 6.

We assessed the effect of DMB on plasma insulin and glucose levels because insulin and glucose levels can influence brain injury. GLP-1R agonists have the potential to stimulate insulin secretion in a glucose-dependent manner. It is rational to speculate that DMB had no impact on plasma insulin and glucose level and we can take it as the expected result, but actually there is no data on the effect of DMB in normal animals. Therefore, the effect of DMB on plasma insulin and glucose levels was determined in normal mice. Results showed no significant differences in plasma insulin (Fig 5A) and blood glucose (Fig 5B) levels after administration of DMB (5 μmol/kg, orally). These two parameters were stable in the mice during the 120 min treatment period.

### 5. DMB improved functional outcomes and reduced infarct size in stroke model

**Fig 6:**
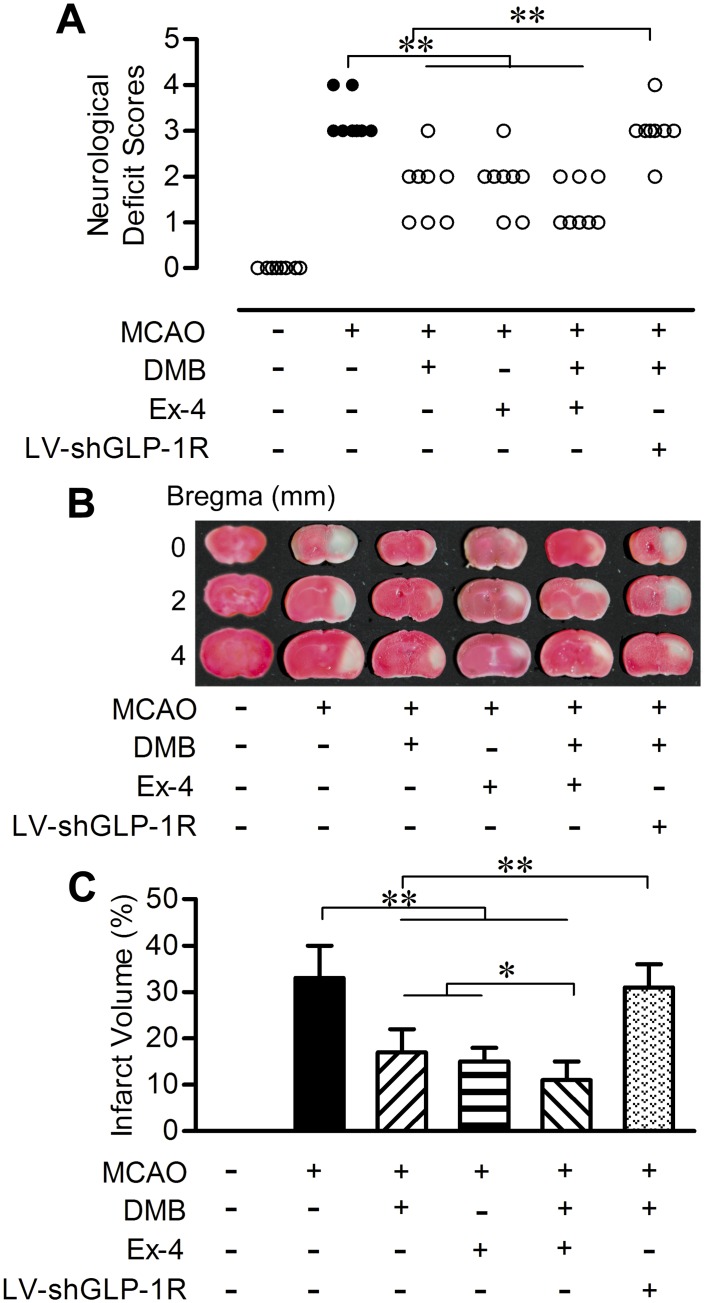
DMB treatment improved neurological deficit and decreased infarct volume 24 h after MCAO. (A) Neurological deficit scores. n = 8. (B) Photographs of brain slices stained with TTC. (C) Quantitative analysis of infarct volume. n = 6. * *p* < 0.05, ** *p* < 0.01.

We further evaluated the neuroprotective effects of DMB on cerebral ischemia in MCAO mice by comparing neurological deficits and infarct volume. MCAO injury caused an obvious increase in the neurological deficit score. Oral administration of DMB (5 μmol/kg), intraperitoneal injection of exendin-4 (10 nmol/kg) as well as the combination of these two drugs significantly decreased the neurological deficit caused by MCAO. The effect of DMB was reversed by intracerebroventricular infusion of LV-shGLP-1R (Fig 6A).

The infarct volume assessed using TTC staining was in accordance with the neurological deficit scores. Oral administration of DMB (5 μmol/kg) and intraperitoneal injection of exendin-4 (10 nmol/kg) reduced the infarct volume caused by MCAO. The combination of DMB (5 μmol/kg, oral) and exendin-4 (10 nmol/kg, i.p.) further improved the beneficial efficacy. Silencing of GLP-1R by LV-shGLP-1R, however, diminished the neuroprotective effects of DMB (Fig 6B and 6C).

### 6. DMB stimulated the cAMP-PKA-CREB signaling pathway after MCAO

**Fig 7:**
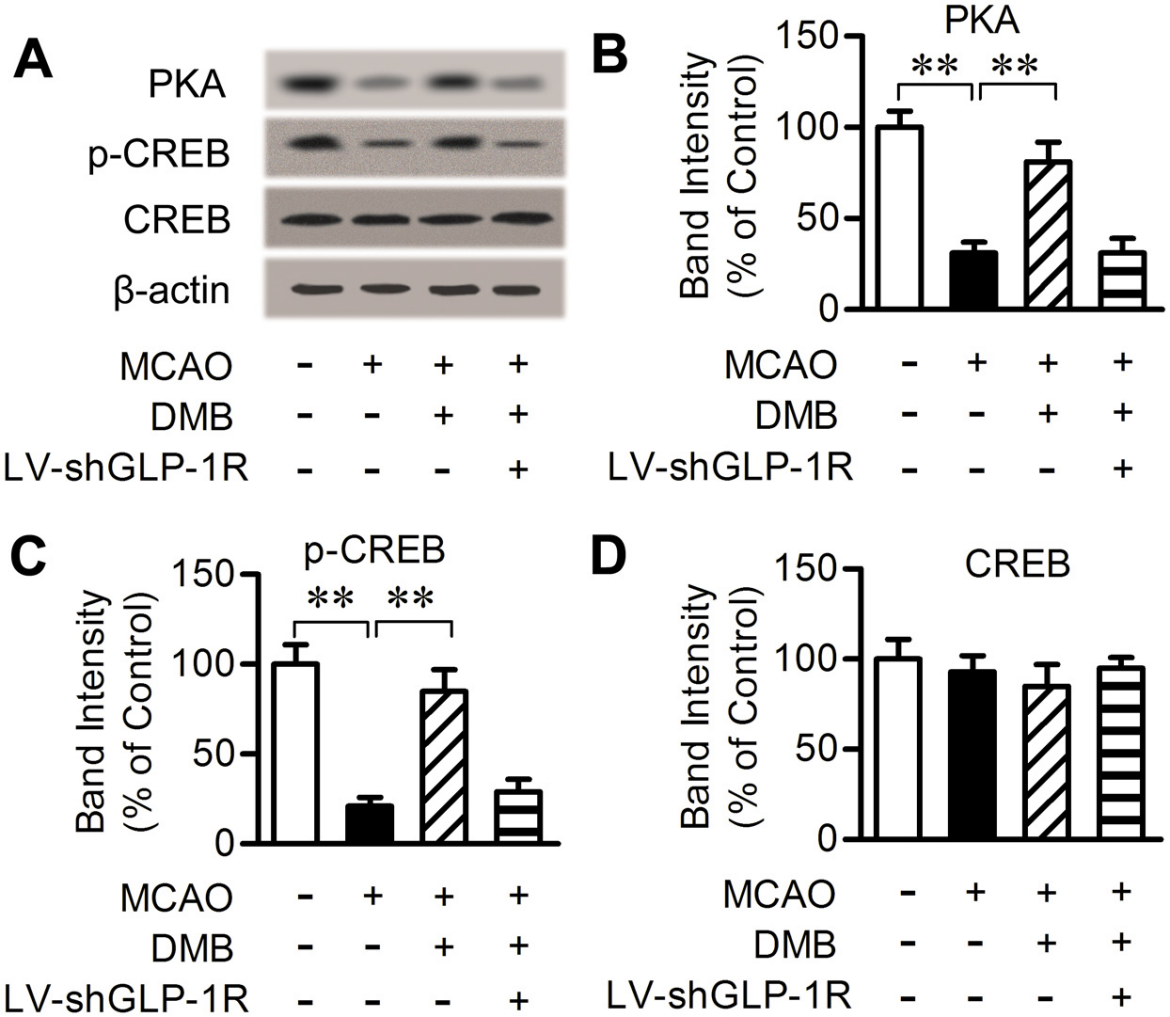
Effects of DMB on the cAMP-PKA-CREB signaling pathway after MCAO. (A) Western blot analysis of PKA, p-CREB and CREB in ipsilateral ischemic penumbra 24 h post MCAO or sham operation. (B-D) Bar graphs reflect PKA, p-CREB and CREB proteins in each group. Data are expressed as a percentage of sham. n = 6. ** *p* < 0.01.

cAMP signaling leads to phosphorylation and activation of CREB through PKA, stimulating gene expression that promotes neuronal viability [27–29]. Thus, the effect of DMB on this signaling pathway after MCAO was evaluated. Results show that PKA and p-CREB (Fig 7A–7C) levels were significantly decreased after MCAO injury in the ischemic penumbra area. These trends were reversed by pretreatment with DMB (5 μmol/kg). This effect, however, was diminished by intracerebroventricular infusion of LV-shGLP-1R. The presence or absence of MCAO and DMB had no impact on the expression of total CREB levels (Fig 7A and 7D). These results indicate that DMB stimulates the cAMP-PKA-CREB signaling pathway via activation of the GLP-1R.

### 7. DMB mediated anti-apoptotic effect against MCAO injury

**Fig 8:**
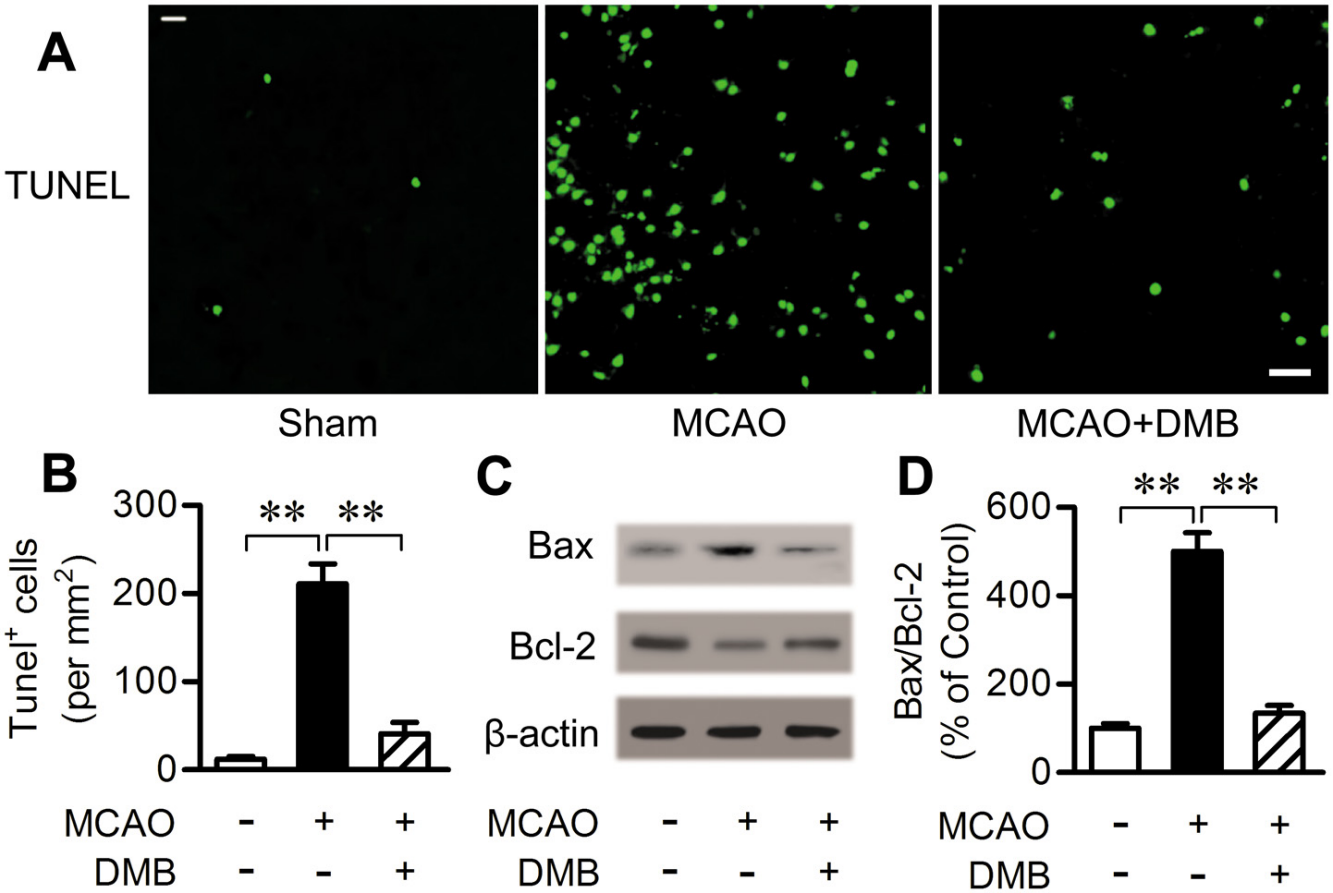
DMB treatment decreased neuronal apoptosis in cerebral ischemia. (A) TUNEL assay 24 h post MCAO or sham operation. Photographs were taken from ipsilateral ischemic penumbra area. Scale bar = 25 μm. (B) Bar graph reflects the number of TUNEL-positive neurons in each group. Six different fields were counted. (C) Western blot assay of Bax and Bcl-2 expression in ipsilateral ischemic penumbra area 24 h post MCAO or sham operation. (D) Bar graph reflects Bax/Bcl-2 ratio in each group. n = 6. ** *p* < 0.01.

Apoptosis is suggested to be one of the leading contributors to neuronal injury after cerebral ischemia [30, 31]. Neuron apoptosis in ipsilateral ischemic penumbra was evaluated by TUNEL staining, a specific staining of DNA fragmentation which results from apoptotic signaling cascades. Only a few TUNEL-positive cells were observed in the sham group; however, MCAO caused a significant increase in TUNEL-positive cells 24 h after injury. In contrast, DMB (5 μmol/kg, orally) significantly decreased the number of TUNEL-positive cells compared with the MCAO group (Fig 8A and 8B).

Bcl-2 and Bax play an important role in the regulation of neuronal apoptosis. Bcl-2 is an anti-apoptotic protein while Bax can promote apoptosis. Western blot results showed a strong downregulation of Bcl-2 and upregulation of Bax 24 h after cerebral ischemia in the ipsilateral ischemic penumbra area. This trend, however, was reversed by oral administration of DMB (5 μmol/kg) (Fig 8C and 8D). These results indicate that DMB has an anti-apoptotic effect against cerebral ischemia.

## Discussion

GLP-1 can be released from L cells following the ingestion of food. GLP-1 exerts many biological effects by activating the GLP-1R signaling pathway, including the induction of glucose-dependent insulin release, inhibition of glucagon secretion and stimulation of β-cell proliferation [32]. As previously stated, GLP-1 based drugs have proven to be beneficial for the treatment of T2D.

The GLP-1R belongs to the class B family of G-protein coupled receptors (GPCRs). Upon GLP-1R activation, the major signaling pathway involves activation of adenylyl cyclase via stimulatory Gα, leading to an increase in intracellular cAMP levels and a series of subsequent signaling events that regulate various cell functions [33]. The GLP-1R is distributed in many peripheral tissues (e.g. pancreatic islets, heart, kidney, and gastrointestinal tract) and is also widely expressed in many regions of the brain [32, 33]. Recent research has showed neuroprotective properties against ischemic stroke by drugs targeting GLP-1R [4, 5, 11–15]. Therefore, the GLP-1R is emerging as a new therapeutic target for the treatment of cerebral ischemic stroke.

Diabetes is believed to be a generally accepted risk factor related with episodes of stroke [16]. GLP-1R agonists are in clinical use for the treatment of diabetes and several studies indicate that they may be useful in the treatment of ischemic stroke, but the neuroprotective efficiency of orally administered low molecular weight agonists has not been reported. It is difficult to identify nonpeptide, low molecular weight GLP-1R agonists that are orally active. GLP-1R activation by peptide agonists requires binding predominantly to the large N-terminal domain of the receptor [34]. It is difficult for the surrogate small molecule agonists to mimic this action due to the size of the peptide ligands and their mechanism for receptor activation. Small ligands may not be able to fully mimic the actions of larger peptide agonists. Nonetheless, several small molecules have been successfully identified as GLP-1R agonists, including DMB and Boc-5—a large cyclobutane derivative.

The GLP-1R is comprised of eight hydrophobic domains, seven of which span the membrane, with a further extracellular N-terminal domain. GLP-1 and its peptide agonists bind to the receptor in an orthosteric manner. Recently published data reported that a range of polyclonal anti-GLP-1R antibodies cannot be used to detect GLP-1R in immunohistochemistry or western blotting [35–37] and with no validation of antibody specificity in paraffin-embedded tissues. Although these findings revealed substantial problems with the sensitivity and specificity of antibody against GLP-1R used for the detection of the GLP-1R in various tissues, there are still a lot of researches performed by western blot analysis to detect the expression of GLP-1R [38–40]. We found that primary cortical neurons expressed GLP-1R at protein levels, and then incubation of these cells with the DMB caused a dramatic increase in the intracellular cAMP levels, demonstrating the presence of functional GLP-1R in cortical neurons.

However, DMB reportedly interacts with the GLP-1R at an allosteric site that has been speculated to be a cavity located near the transmembrane 5 and 6 region [41]. Exendin (9–39) blocked the effects of GLP-1 by competitively binding the extracellular domain of the GLP-1R [24, 42]. Thus, the cAMP generated by DMB was not inhibited by exendin (9–39). DMB has been shown to potentially increase the affinity of peptide agonists [25]. Here, we show similar results in which the neuroprotective effects of DMB were blocked by GLP-1R knockdown with shRNA but not by a GLP-1R antagonist. Moreover, DMB activated the GLP-1R—leading to increased intracellular cAMP levels in cultured cortical neurons with an EC_50_ value approximately 100 fold compared with exendin-4.

Neuroprotective strategies aimed at decreasing brain damage after ischemic stroke have failed to be translated into the clinical setting along the past decades [43]. Up to now, tPA is still the only approved pharmacological treatment for ischemic stroke. Although most of the current therapies are focused on posttreatment after cerebral ischemia, accumulating lines of evidence have demonstrated the efficacy of pretreatment therapies which could induce neuroprotection against cerebral ischemic injury. GLP-1R agonist exendin-4 is in clinical use for the treatment of diabetes and several studies indicated that it may be useful in the treatment of stroke [4, 5, 11–14]. Li *et al*. found that intracerebral administration of exendin-4 induced neuroprotection and locomotor activity after stroke in the rat, and showed this neuroprotective effect was mediated by GLP-1R in GLP-1R knockout mice [4]. However, the findings have low clinical relevance since exendin-4 was given at stroke onset and via a route of administration not suitable for clinical applications. Teramoto *et al*. showed that transvenous administratration of exendin-4 provides neuroprotection against ischemic injury in mice at 1 h after MCAO, but the effect was lost at 3 h [5]. Another study by Darsalia et al. show neuroprotective efficacy of 50 μg/kg exendin-4 at 1.5 h and 3 h after stroke in both young healthy and aged diabetic/obese mice, and the 5 μg/kg dose was neuroprotective at 1.5 h only [11]. Furthermore, other experiments have demonstrated that administration of exendin-4 before MCAO resulted in neuroprotection [4, 12–14]. Darsalia *et al*. that 4 weeks intraperitoneal pretreatment with exendin-4 before MCAO followed by another 4 weeks of exendin-4 treatment decreases brain damage in T2D rats [14]. GLP-1R agonists have been used in clinical for the treatment of T2D, hyperglycemia is one of the leading risk factors for ischemic cerebrovascular diseases [16]. So, it provided a potential clinical use of GLP-1R agonists for the treatment of stroke in T2D patients or individuals at high risk to suffer from a stroke (e.g. pretreatment strategies). Therefore, the pretreatment strategy was used in our current study based on these previous experiments. Data showed that oral administration of DMB with the therapeutic dose reduced cerebral infarct volume and neurological deficits caused by MCAO.

Stimulation of cAMP can trigger phosphorylation of CREB at serine 133, which plays an important role in neuronal development, synaptic plasticity, and memory formation. Phosphorylation of CREB leads to the expression of genes related to neuroprotection against ischemic insults [27–29]. We found that DMB markedly reduced the cerebral infarct size and neurological deficits caused by MCAO and reperfusion. The neuroprotective effect of DMB was mediated by activation of the GLP-1R through the cAMP-PKA-CREB signaling pathway. Activation of the GLP-1R resulted in an improvement of glycemic control, which could lower blood glucose and stimulate insulin release. Glucose level can influence brain injury. Insulin was reported to confer a neuroprotective effect in cerebral ischemia in MCAO [44]. Exendin-4 was reported to confer neuroprotective effect in a MCAO model of diabetic rats [14]. Thus, the peripheral effect on hyperglycemia induced by DMB is necessary to be excluded in non-diabetic mice. Since the level of blood glucose and plasma insulin was stable before and after DMB administration, and knock-down of the GLP-1R in the brain blocked the neuroprotective effect, we can conclude that DMB conferred the neuroprotective effect by activating GLP-1R in the brain.

These results demonstrate that DMB, a small molecular weight GLP-1R agonist, attenuates transient focal cerebral ischemia injury and inhibits neuronal apoptosis *in vivo* and *in vitro*. Therefore, DMB represents a potential neuroprotective agent due to its ability to act alone or in combination with other peptide GLP-1R receptor agonists.

## Supporting Information

S1 Fig LV-shGLP-1R silenced GLP-1R expression in cultured neurons and ipsilateral ischemic penumbra. (A) Western blot analysis of GLP-1R in neurons. (B) Western blot analysis of GLP-1R in ipsilateral ischemic penumbra area.
(TIF)

## References

[1] Go AS, Mozaffarian D, Roger VL, Benjamin EJ, Berry JD, Blaha MJ, et al Executive summary: heart disease and stroke statistics—2014 update: a report from the American Heart Association. Circulation. 2014;129(3):399–410. 10.1161/01.cir.0000442015.53336.12 . 24446411

[2] Kieffer TJ, Habener JF. The glucagon-like peptides. Endocr Rev. 1999;20(6):876–913. 10.1210/edrv.20.6.0385 . 10605628

[3] Thorens B. Expression cloning of the pancreatic beta cell receptor for the gluco-incretin hormone glucagon-like peptide 1. Proceedings of the National Academy of Sciences of the United States of America. 1992;89(18):8641–5. 1326760 10.1073/pnas.89.18.8641 PMC49976

[4] Li Y, Perry T, Kindy MS, Harvey BK, Tweedie D, Holloway HW, et al GLP-1 receptor stimulation preserves primary cortical and dopaminergic neurons in cellular and rodent models of stroke and Parkinsonism. Proceedings of the National Academy of Sciences of the United States of America. 2009;106(4):1285–90. 10.1073/pnas.0806720106 19164583 PMC2633544

[5] Teramoto S, Miyamoto N, Yatomi K, Tanaka Y, Oishi H, Arai H, et al Exendin-4, a glucagon-like peptide-1 receptor agonist, provides neuroprotection in mice transient focal cerebral ischemia. Journal of cerebral blood flow and metabolism: official journal of the International Society of Cerebral Blood Flow and Metabolism. 2011;31(8):1696–705. 10.1038/jcbfm.2011.51 21487412 PMC3170947

[6] Darsalia V, Larsson M, Nathanson D, Klein T, Nystrom T, Patrone C. Glucagon-like receptor 1 agonists and DPP-4 inhibitors: potential therapies for the treatment of stroke. Journal of cerebral blood flow and metabolism: official journal of the International Society of Cerebral Blood Flow and Metabolism. 2015;35(5):718–23. 10.1038/jcbfm.2015.17 25669907 PMC4420864

[7] Holscher C. Potential role of glucagon-like peptide-1 (GLP-1) in neuroprotection. CNS drugs. 2012;26(10):871–82. 10.2165/11635890-000000000-00000 . 22938097

[8] Deacon CF, Nauck MA, Toft-Nielsen M, Pridal L, Willms B, Holst JJ. Both subcutaneously and intravenously administered glucagon-like peptide I are rapidly degraded from the NH2-terminus in type II diabetic patients and in healthy subjects. Diabetes. 1995;44(9):1126–31. . 7657039 10.2337/diab.44.9.1126

[9] Manandhar B, Ahn JM. Glucagon-like Peptide-1 (GLP-1) Analogs: Recent Advances, New Possibilities, and Therapeutic Implications. Journal of medicinal chemistry. 2014 10.1021/jm500810s . 25349901 PMC4329993

[10] Tzefos M, Harris K, Brackett A. Clinical efficacy and safety of once-weekly glucagon-like peptide-1 agonists in development for treatment of type 2 diabetes mellitus in adults. The Annals of pharmacotherapy. 2012;46(1):68–78. 10.1345/aph.1Q379 . 22232377

[11] Darsalia V, Hua S, Larsson M, Mallard C, Nathanson D, Nystrom T, et al Exendin-4 reduces ischemic brain injury in normal and aged type 2 diabetic mice and promotes microglial M2 polarization. PloS one. 2014;9(8):e103114 10.1371/journal.pone.0103114 25101679 PMC4125154

[12] Briyal S, Gulati K, Gulati A. Repeated administration of exendin-4 reduces focal cerebral ischemia-induced infarction in rats. Brain research. 2012;1427:23–34. 10.1016/j.brainres.2011.10.026 . 22055454

[13] Lee CH, Yan B, Yoo KY, Choi JH, Kwon SH, Her S, et al Ischemia-induced changes in glucagon-like peptide-1 receptor and neuroprotective effect of its agonist, exendin-4, in experimental transient cerebral ischemia. Journal of neuroscience research. 2011;89(7):1103–13. 10.1002/jnr.22596 . 21472764

[14] Darsalia V, Mansouri S, Ortsater H, Olverling A, Nozadze N, Kappe C, et al Glucagon-like peptide-1 receptor activation reduces ischaemic brain damage following stroke in Type 2 diabetic rats. Clinical science. 2012;122(10):473–83. 10.1042/CS20110374 22150224 PMC3268352

[15] Sato K, Kameda M, Yasuhara T, Agari T, Baba T, Wang F, et al Neuroprotective effects of liraglutide for stroke model of rats. International journal of molecular sciences. 2013;14(11):21513–24. 10.3390/ijms141121513 24177570 PMC3856019

[16] Tanne D. Impaired glucose metabolism and cerebrovascular diseases. Advances in cardiology. 2008;45:107–13. 10.1159/0000115190 . 18230958

[17] Meloni BP, Majda BT, Knuckey NW. Establishment of neuronal in vitro models of ischemia in 96-well microtiter strip-plates that result in acute, progressive and delayed neuronal death. Neuroscience. 2001;108(1):17–26. . 11738128 10.1016/s0306-4522(01)00396-7

[18] Zhang Z, Yan J, Taheri S, Liu KJ, Shi H. Hypoxia-inducible factor 1 contributes to N-acetylcysteine's protection in stroke. Free Radic Biol Med. 2014;68:8–21. 10.1016/j.freeradbiomed.2013.11.007 24296245 PMC3943875

[19] Dong YF, Wang LX, Huang X, Cao WJ, Lu M, Ding JH, et al Kir6.1 knockdown aggravates cerebral ischemia/reperfusion-induced neural injury in mice. CNS neuroscience & therapeutics. 2013;19(8):617–24. 10.1111/cns.12117 . 23663330 PMC6493342

[20] Bederson JB, Pitts LH, Germano SM, Nishimura MC, Davis RL, Bartkowski HM. Evaluation of 2,3,5-triphenyltetrazolium chloride as a stain for detection and quantification of experimental cerebral infarction in rats. Stroke. 1986;17(6):1304–8. . 2433817 10.1161/01.str.17.6.1304

[21] Zhang H, Meng J, Li X, Zhou S, Qu D, Wang N, et al Pro-GLP-1, a Pro-drug of GLP-1, is neuroprotective in cerebral ischemia. Eur J Pharm Sci. 2015;70:82–91. 10.1016/j.ejps.2015.01.010 . 25640912

[22] Chen T, Wang J, Li C, Zhang W, Zhang L, An L, et al Nafamostat mesilate attenuates neuronal damage in a rat model of transient focal cerebral ischemia through thrombin inhibition. Scientific reports. 2014;4:5531 10.1038/srep05531 24985053 PMC4078306

[23] Goke R, Fehmann HC, Linn T, Schmidt H, Krause M, Eng J, et al Exendin-4 is a high potency agonist and truncated exendin-(9–39)-amide an antagonist at the glucagon-like peptide 1-(7–36)-amide receptor of insulin-secreting beta-cells. The Journal of biological chemistry. 1993;268(26):19650–5. . 8396143

[24] Thorens B, Porret A, Buhler L, Deng SP, Morel P, Widmann C. Cloning and functional expression of the human islet GLP-1 receptor. Demonstration that exendin-4 is an agonist and exendin-(9–39) an antagonist of the receptor. Diabetes. 1993;42(11):1678–82. . 8405712 10.2337/diab.42.11.1678

[25] Knudsen LB, Kiel D, Teng M, Behrens C, Bhumralkar D, Kodra JT, et al Small-molecule agonists for the glucagon-like peptide 1 receptor. Proceedings of the National Academy of Sciences of the United States of America. 2007;104(3):937–42. 10.1073/pnas.0605701104 17213325 PMC1783418

[26] Camos S, Mallolas J. Experimental models for assaying microvascular endothelial cell pathophysiology in stroke. Molecules. 2010;15(12):9104–34. 10.3390/molecules15129104 . 21150829 PMC6259215

[27] Wu F, Echeverry R, Wu J, An J, Haile WB, Cooper DS, et al Tissue-type plasminogen activator protects neurons from excitotoxin-induced cell death via activation of the ERK1/2-CREB-ATF3 signaling pathway. Mol Cell Neurosci. 2013;52:9–19. 10.1016/j.mcn.2012.10.001 23063501 PMC3540185

[28] Ashabi G, Khodagholi F, Khalaj L, Goudarzvand M, Nasiri M. Activation of AMP-activated protein kinase by metformin protects against global cerebral ischemia in male rats: interference of AMPK/PGC-1alpha pathway. Metab Brain Dis. 2014;29(1):47–58. 10.1007/s11011-013-9475-2 . 24435937

[29] Nakka VP, Gusain A, Raghubir R. Endoplasmic reticulum stress plays critical role in brain damage after cerebral ischemia/reperfusion in rats. Neurotox Res. 2010;17(2):189–202. 10.1007/s12640-009-9110-5 . 19763736

[30] MacManus JP, Buchan AM. Apoptosis after experimental stroke: fact or fashion? J Neurotrauma. 2000;17(10):899–914. . 11063056 10.1089/neu.2000.17.899

[31] Sairanen T, Karjalainen-Lindsberg ML, Paetau A, Ijas P, Lindsberg PJ. Apoptosis dominant in the periinfarct area of human ischaemic stroke—a possible target of antiapoptotic treatments. Brain. 2006;129(Pt 1):189–99. 10.1093/brain/awh645 . 16272167

[32] Baggio LL, Drucker DJ. Biology of incretins: GLP-1 and GIP. Gastroenterology. 2007;132(6):2131–57. 10.1053/j.gastro.2007.03.054 . 17498508

[33] Perry T, Greig NH. The glucagon-like peptides: a double-edged therapeutic sword? Trends Pharmacol Sci. 2003;24(7):377–83. 10.1016/S0165-6147(03)00160-3 . 12871671

[34] Hoare SR. Mechanisms of peptide and nonpeptide ligand binding to Class B G-protein-coupled receptors. Drug Discov Today. 2005;10(6):417–27. . 15808821 10.1016/S1359-6446(05)03370-2

[35] Pyke C, Heller RS, Kirk RK, Orskov C, Reedtz-Runge S, Kaastrup P, et al GLP-1 receptor localization in monkey and human tissue: novel distribution revealed with extensively validated monoclonal antibody. Endocrinology. 2014;155(4):1280–90. 10.1210/en.2013-1934 . 24467746

[36] Panjwani N, Mulvihill EE, Longuet C, Yusta B, Campbell JE, Brown TJ, et al GLP-1 receptor activation indirectly reduces hepatic lipid accumulation but does not attenuate development of atherosclerosis in diabetic male ApoE(-/-) mice. Endocrinology. 2013;154(1):127–39. 10.1210/en.2012-1937 . 23183176

[37] Pyke C, Knudsen LB. The glucagon-like peptide-1 receptor—or not? Endocrinology. 2013;154(1):4–8. 10.1210/en.2012-2124 . 23267050

[38] Wu Z, Liu S, Nair I, Omori K, Scott S, Todorov I, et al (64)Cu labeled sarcophagine exendin-4 for microPET imaging of glucagon like peptide-1 receptor expression. Theranostics. 2014;4(8):770–7. 10.7150/thno.7759 24955138 PMC4063975

[39] Green CJ, Henriksen TI, Pedersen BK, Solomon TP. Glucagon like peptide-1-induced glucose metabolism in differentiated human muscle satellite cells is attenuated by hyperglycemia. PloS one. 2012;7(8):e44284 10.1371/journal.pone.0044284 22937169 PMC3429413

[40] Ding L, Zhang J. Glucagon-like peptide-1 activates endothelial nitric oxide synthase in human umbilical vein endothelial cells. Acta pharmacologica Sinica. 2012;33(1):75–81. 10.1038/aps.2011.149 22120969 PMC4010269

[41] Lin F, Wang R. Molecular modeling of the three-dimensional structure of GLP-1R and its interactions with several agonists. Journal of molecular modeling. 2009;15(1):53–65. 10.1007/s00894-008-0372-2 . 18941807

[42] Lopez de Maturana R, Willshaw A, Kuntzsch A, Rudolph R, Donnelly D. The isolated N-terminal domain of the glucagon-like peptide-1 (GLP-1) receptor binds exendin peptides with much higher affinity than GLP-1. The Journal of biological chemistry. 2003;278(12):10195–200. 10.1074/jbc.M212147200 . 12524435

[43] Turner RC, Dodson SC, Rosen CL, Huber JD. The science of cerebral ischemia and the quest for neuroprotection: navigating past failure to future success. Journal of neurosurgery. 2013;118(5):1072–85. 10.3171/2012.11.JNS12408 . 23331000 PMC4652647

[44] Sanderson TH, Kumar R, Murariu-Dobrin AC, Page AB, Krause GS, Sullivan JM. Insulin activates the PI3K-Akt survival pathway in vulnerable neurons following global brain ischemia. Neurological research. 2009;31(9):947–58. 10.1179/174313209X382449 . 19203442

