# Supplementary figures and images for: An Orally Active Allosteric GLP-1 Receptor Agonist Is Neuroprotective in Cellular and Rodent Models of Stroke
Source: PLoS One. 2016 Feb 10;11(2):e0148827. doi: 10.1371/journal.pone.0148827 (PMC4749391; doi:10.1371/journal.pone.0148827)

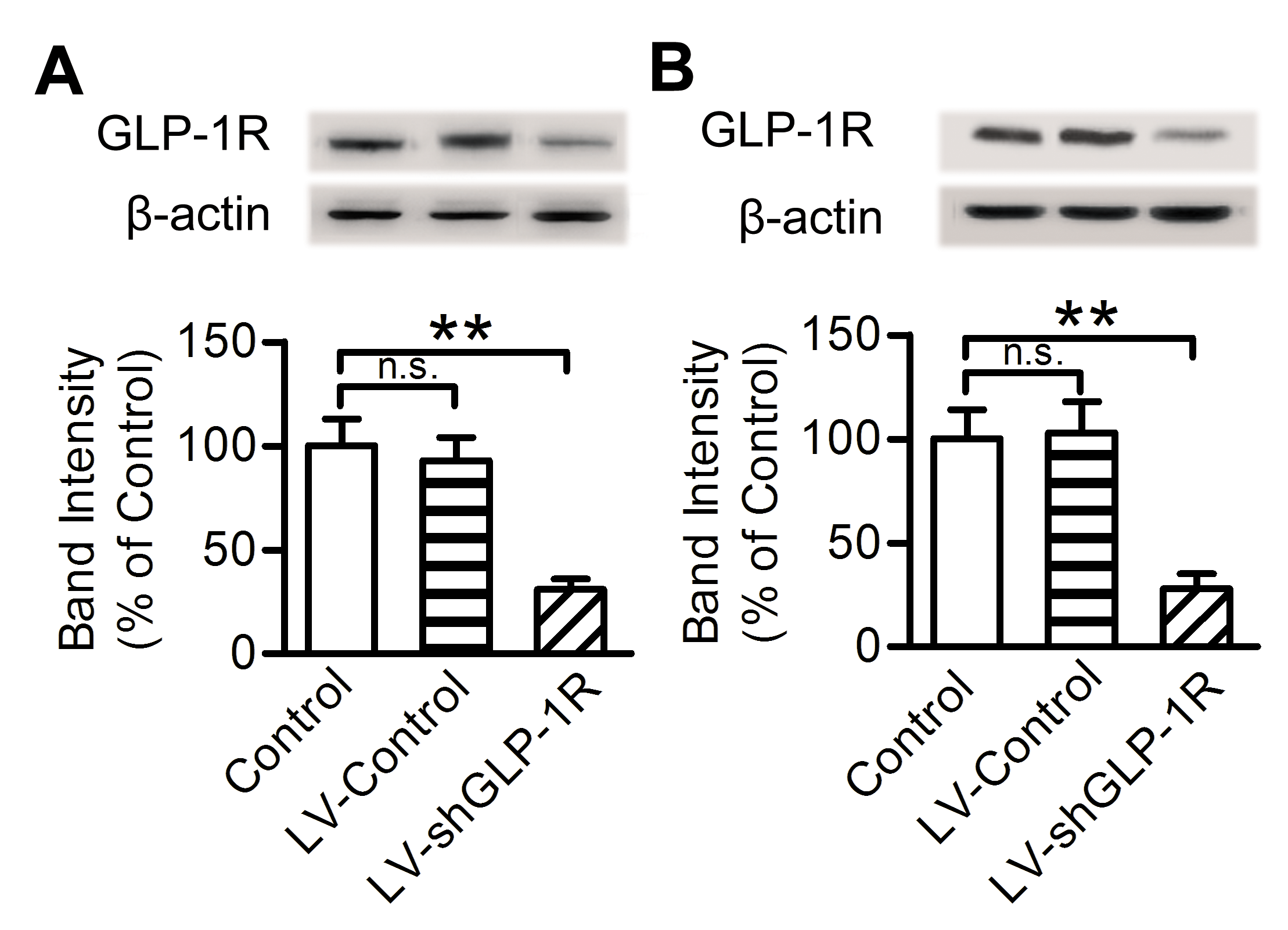

Supplement: S1 Fig — (A) Western blot analysis of GLP-1R in neurons. (B) Western blot analysis of GLP-1R in ipsilateral ischemic penumbra area. (TIF) [file pone.0148827.s001.tif]
